# Bioactive Chemical Constituents and Pharmacological Activities of Ponciri Fructus

**DOI:** 10.3390/molecules28010255

**Published:** 2022-12-28

**Authors:** Gopal Lamichhane, Jitendra Pandey, Hari Prasad Devkota

**Affiliations:** 1 Department of Oriental Pharmacy and Wonkwang-Oriental Medicines Research Institute, Wonkwang University, Iksan 570-749, Republic of Korea; 2 Department of Pharmacy, Crimson College of Technology, Pokhara University, Devinagar-11, Butwal 32900, Nepal; 3 Graduate School of Pharmaceutical Sciences, Kumamoto University, 5-1 Oe-honmachi, Chuo-ku, Kumamoto 862-0973, Japan; 4 Headquarters for Admissions and Education, Kumamoto University, Kurokami, 2-39-1, Chuo-ku, Kumamoto 860-8555, Japan; 5 Pharmacy Program, Gandaki University, Pokhara 33700, Nepal

**Keywords:** *Poncirus trifoliata*, ponciri Fructus, orange trifoliate, ethnomedicine, anti-obesity, phytochemistry, immature fructus

## Abstract

Ponciri Fructus is a crude drug obtained from the dried immature fruits of *Poncirus trifoliata* (L). Raf. (Syn. *Citrus trifoliata* L.). This study aims to compile and analyze the ethnomedicinal uses, bioactive constituents, and pharmacological activities of Ponciri Fructus. Various online bibliographic databases namely, SciFinder, PubMed, Google Scholar, and Web of Science were used for collecting information on traditional uses, biological activities, and bioactive constituents. Concerning ethnomedicinal uses, Ponciri Fructus is extensively used in traditional Korean, Chinese, and Kampo medicines to mitigate allergic reactions, inflammation, edema, digestive complications, respiratory problems, spleen-related problems, liver complications, neuronal pain, hyperlipidemia, rheumatoid arthritis, cardiovascular problems, hernia, sinusitis, and insomnia. Several studies have shown that Ponciri Fructus is a major source of diverse classes of bioactive compounds namely flavonoids, terpenoids, coumarins, phytosterols, and alkaloids. Several in vivo and in vitro pharmacological activity evaluations such as antidiabetic, anti-obesity, anti-inflammatory, antiallergic, antimelanogenic, gastroprotective, anticancer, and neuroprotective effects have been conducted from Ponciri Fructus. However, scientific investigations focusing on bioassay-guided isolation and identification of specific bioactive constituents are limited. Therefore, an in-depth scientific investigation of Ponciri Fructus focusing on bioassay-guided isolation, mechanism based pharmacological studies, pharmacokinetic studies, and evaluation of possible toxicities is necessary in the future.

## 1. Introduction

Plants possessing medicinal properties have been playing a crucial role in maintaining human health via their multiple utilizations as conventional medicines, spices, and food, and as a paramount source of the lead molecule during novel drug discovery and development [1,2]. In current years, interest in plant-derived functional foods and nutraceuticals has been increasing tremendously in order to protect human beings from numerous lifestyle-associated diseases and several other health issues such as atherosclerosis [3], inflammation [4,5], endothelial dysfunction [6], cancer progression [7,8,9], hypercholesterolemia [10], and asthma [11]. Diverse types of plant-derived nutritional constituents such as vitamins, minerals, and amino acids, as well as bioactive phytoconstituents such as polyphenols (flavonoids and phenolic acids), are gaining increased interest [12,13,14]. In addition, various plant-based products are being manufactured as edible formulations. This is despite the fact that in order to identify the exact chemical structure of bioactive molecules present in these plants, an in-depth investigation of the pharmacological actions and an outline of worthwhile drug delivery system and formulations are crucial and challenging scientific tasks that have to be performed [7].

**Figure 1:**
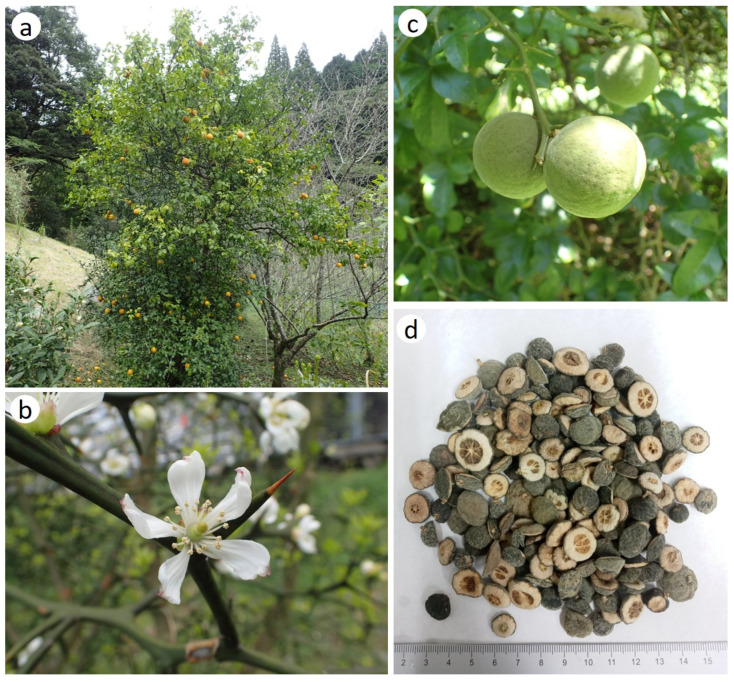
Photographs of different plant parts of *Poncirus trifoliata*. (**a**) Tree bearing mature fruits, (**b**) flower (**c**) immature fruits and (**d**) dried immature fruits used as crude drug Ponciri Fructus. [Photo credits: Masato Watanabe (**a**–**c**), Gopal Lamichhane (**d**)].

Ponciri Fructus (Figure 1) is a dried immature fruit of *P. trifoliata* (L). Raf. (Syn. *Citrus trifoliata* L.) is commonly known as Trifoliate Orange, Japanese Bitter Orange, or Chinese Bitter Orange. It is a deciduous or semi-deciduous prickly shrub belonging to the family Rutaceae and is widely distributed in temperate regions of East Asian countries such as South Korea, North Korea, China, and Japan with a height of 3.5 m. Although recent plant databases include this plant in the genus *Citrus*, some other classify it into the genus *Poncirus* as this plant possesses some distinguishable features such as deciduous distribution, pubescent fruits, and compound leaves [15,16]. Only two species i.e., *P. trifoliata* and *P. polyandra* have been recognized from this genus [17]. In South Korea, *P. trifoliata* is native to Gaduk and Jeju Island [18]. Nowadays, it is being cultivated commercially in the central and southern regions of Korea [18,19]. This species is an extremely cold-resistant plant and is cross-compatible with other subtropical citrus plants. For this reason, in the temperate regions of many countries such as Japan, China, New Zealand, and Australia, it has been utilized as a rootstock to graft other citrus fruits. In addition, citrus plants in this rootstock can produce high-yield and quality fruits [17,20]. The most applicable part of this plant is pubescent fruit (3–4 cm in diameter) with greenish pulp, which looks like a small orange and gives a pleasing fragrance. The fruit changes from green to yellow or golden color, upon maturity [15,21,22]. This fruit is called ‘Jisil’ (in Korean) and Zhishi (in Chinese), is a well-known oriental medicine for management of allergic diseases [23,24]. Another characteristic feature of this plant is the presence of distinctive leaves which are formed by the fusion of oval-shaped three-lobed leaflets where the central leaflet is slightly larger. These leaves are scarce, glossy, and leathery in appearance. The most aromatic parts of this plant are the white-colored five-flowered petals. In the middle of the spring season, the plant starts to bloom before the appearance of leaves, and fruits ripen completely in the late fall. The harvesting time of mature fruits is from May to June [19]. The fresh fruits of this plant are not suitable for oral consumption due to the presence of numerous seeds, the low quantity of pulp, and its unpleasant taste. Therefore, fruits are dried and comminuted into fine powder for consumption as a condiment. Nowadays, the fermentation of fresh fruit is very popular, as it can reduce the bitter taste and improve the nutritional value of fruit [16].

## 2. Cultivation of *P. trifoliata*

Most often, *P. trifoliata* is cultivated either by directly sowing the seeds in the spring season or through cutting propogation in the summer season. The seeds are greatly resistant to extremely cold environments up to −30 °C but intolerant to drought conditions. Normally, seeds are stratified in a cold environment for four weeks, before sowing in a greenhouse in the early spring. After two weeks, seedlings are plucked and transferred into individual pots and filled with acid soil (pH 4) inside the greenhouse. The relative humidity of the greenhouse should be in the range of 40–80%. It is necessary to let them grow in the pot for no less than their first winter. After that, plants are transferred to permanent places near to the summer season. These plants prefer a sunny area with well-drained sandy, acidic soil for their proper growth. In addition, the supply of water should be comparatively high during the fruiting season of this plant [25,26]. Moreover, other sophisticated techniques such as tissue sub-culturing [27], rootstock grafting [28], seed inoculation with arbuscular mycorrhizal fungi [29,30], etc., are gaining popularity in the commercial production of this plant.

## 3. Traditional Uses of Ponciri Fructus

In the traditional Chinese and Korean systems of medicine, the dried immature fruit has been used to mitigate allergic reactions [23,31], indigestion, inflammation [23], edema [32], duodenal ulcers, gastric ulcers [33], gastritis, constipation, dysentery, and other digestion related complications [34,35]. In eastern Asian countries, its dried immature fruits are considered an effective remedy to treat hepatotoxicity and inflammation [36]. In traditional Korean and Chinese formulations such as Samchulgeonbi-tang (combination of 14 different crude drugs) [37], Mahwangyounpae-tang (combination of 22 different crude drugs) [38], CGX (combination of 13 different crude drugs) [39,40], Sayuk-san (combination of 4 different crude drugs) [41], and Jeechool-Whan (combination of Ponciri Fructus and Atractylodis Rhizoma Alba) [42], Ponciri Fructus is incorporated as a significant constituent and these formulations are widely used for various complications such as gastric ulcers, indigestion, chronic gastritis, gastroptosis, emesis, and diarrhea [37,41,42], along with several respiratory problems [38], spleen related problems [42], and liver complications (liver cirrhosis, viral hepatitis, jaundice, and alcoholic liver damage) [39,40]. Moreover, another Korean traditional formulation Ojeok-san (a combination of 17 different crude drugs including Ponciri Fructus) has been used as a folk remedy for the mitigation of numerous pathological conditions such as neuronal pain, hyperlipidemia, fever, and rheumatoid arthritis [43,44,45]. Ethnomedicinally, Ponciri Fructus is extensively being used as a prokinetic agent, to improve the abnormal contraction of the uterus, to ameliorate the flow of the blood [18,46,47], and to cure gastroesophageal reflux disease [48]. According to the philosophy of traditional Chinese medicine, Ponciri Fructus can modulate the proper flow of stagnant qi energy, remove the unwanted accumulation of food, eliminate excess mucus, and get rid of unwanted mass formations in the body [49]. Therefore, it is widely used in traditional Chinese medicine to treat chest pain, fullness of the chest, sputum, asthma, and bronchitis [49,50,51,52,53]. As a folk remedy for diverse digestive complications such as abnormality of gastric motility and abnormal GI secretion [54], a usual dose of Ponciri Fructus varies from 2–75 mg [55]. In Korea, Ponciri Fructus extract is incorporated into several allopathic over-the-counter drugs to treat several pathological conditions of the gastrointestinal tract [55,56]. Moreover, dried immature fruits are also widely accepted folk remedies for the complications of hernia, sinusitis, insomnia, and sputum formation [25].

## 4. Bioactive Chemical Constituents

### 4.1. Phytochemical Constituents of Ponciri Fructus

**Figure 2:**
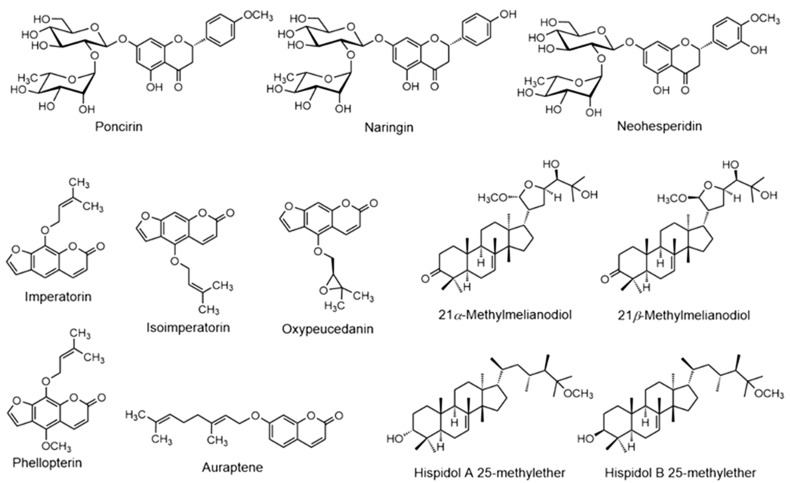
Structure of representative bioactive compounds isolated from Ponciri Fructus.

Although the ethnomedicinal use of *P. trifoliata* has a very long history in Korea, Japan, and China, scientific investigations focused on the in-depth phytochemistry are limited. Even though various parts of the plant such as root, stem bark, leaves, flower, fruits, and seeds are being examined for their bioactive compounds, the majority of the investigations have been focused on immature fruits of this plant. Several studies have shown that the dried immature fruits are a major source of diverse classes of bioactive compounds namely flavonoids, terpenoids, coumarins, phytosterols, and alkaloids [49]. Structure of representative compounds isolated from Ponciri Fructus are presented in Figure 2.

#### 4.1.1. Flavonoids

Flavonoids are the most abundant bioactive constituents present in Ponciri Fructus, and poncirin is the most widely distributed flavonoid compound, which constitutes 6% of the dried fruit extract. Several flavonoid derivatives namely poncirin, neoponcirn, naringin [32,33], hesperidin, neohesperidin, sinensetin, nobiletin, hesperetin [18,47,57], narirutin, naringin 4′-glucoside [16,58], poncirenin, (2*R*)-5-hydroxy-4′-methoxyflavanone-7-*O*-{*β*-glucopyranosyl-(1→2)- *β*-glucopyranoside} [59], and hesperidin methyl chalone [60] have also been discovered from the Ponciri Fructus.

#### 4.1.2. Terpenoids

Terpenoids are largest groups of secondary metabolites widely distributed in plants [61]. From the methanolic extract of Ponciri Fructus, four different bioactive terpenoids 21β-methylmelianodiol, ispidol β 25-methyl ether, hispidol A 25-methyl ether, and 21α-methylmelianodiol have been identified [31]. Similarly, novel terpenoid derivatives (Pancastatin A and B) [62], 25-methoxyhispidol A and B, [51,63] were also reported from the Ponciri Fructus.

#### 4.1.3. Coumarins

Citrus species are among the richest sources of coumarin and its derivatives [64]. Ponciri Fructus methanolic extract was reported to contain diverse coumarin compounds such as isoschininallylol [31], methoxsalen [21], imperatorin [32], isoimperatorin [36,65], phellopterin [65], auraptene [52], umbelliferone [32], phellopterin, oxypeucedanin [66], triphasiol, and ponciol [67]. According to a previous study conducted in South Korea, three potent chemopreventive coumarins: poncimarin, oxypeucedanin methanolate, and heraclenol 30′-methyl ester were isolated from the methylene chloride extract of Ponciri Fructus [68].

#### 4.1.4. Miscellaneous Compounds

Several other subclasses of bioactive compounds such as β-sitosterol (phytosterol) [66], bis(2-methylheptyl) phthalate, avenalumic acid methyl ester (styrene derivative) [69], limonin (furanolactone) [53], synephrine (alkaloid) [70], 2-hydroxyl-1,2,3-propanetricarboxylic acid 2-methyl ester [71] have also been reported from Ponciri Fructus.

### 4.2. Volatile Constituents from the Peel of P. trifoliata Fresh Fruits

In a previous study, essential oils were isolated from the fresh peel of fruits by using hydro-distillation, and volatile chemical constituents present in the oil were identified by GC-MS total ion chromatogram. All the identified essential oils and their relative amounts are depicted in Table 1 [72]. Similarly, another study conducted in China reported two other different volatile components: ocimene and cis-caryophyllene [73] along with 19 other previously identified volatile compounds [72].

### 4.3. Chemical Constituents from the Fresh Fruit Juice and Seed Extract of P. trifoliata

Tundis et al. investigated phytochemicals from the juice of fresh fruits and methanolic extract of the seeds extracts by using HPLC connected with a photodiode array detector. By comparing chromatogram with standard compounds, they found several flavonoid derivatives and phenolic acids in tested samples [69]. The name of the bioactive compounds detected in fruit juice and seed extract are given in Table 2

## 5. Pharmacological Activities

### 5.1. Anti-Obesity Effects

Ban et al. reported the anti-obesity effects of Ponciri Fructus in rats that were fed a high-fat diet (HFD). They found that fruit extract, when fed with a high-fat diet, significantly reduced serum levels of low-density lipoprotein (LDL), fatty acids, and glyceride in rats [74]. Another study on C57BL/6 mice treated with fruit extract also reduced mice weight, fasting blood glucose, liver and adipose tissue weight, serum triglyceride, and LDL significantly [75]. They found that the extract effectively increased expression of carnitine palmitoyltransferase 1α and insulin receptor substrate, modulated serum insulin, adiponectin and leptin expression, while reducing transcription levels of fatty acid synthase and stearoyl-CoA desaturase 1 [75]. Kim et al. found that Ponciri Fructus ameliorated macrophage-mediated inflammation and improved insulin resistance in HFD-fed mice [46]. Shim et al. proposed that the anti-obesity of fruit might be due to its prokinetic effect through the inhibition of nutrient absorption into the blood stream [55]. The absorption might have been hindered due to the inhibition of α-amylase and glucosidase enzymes by this fruit [69]. The observed anti-obesity activity might also be due to the anti-adipogenic effect of fruits as ethanol extract, and hexane and ethyl acetate fraction of this fruit extract significantly reduced adipogenesis in 3T3-L1 preadipocytes [66]. The extract was found to contain poncirin, phellopterin, oxypeucedanin as potent anti-adipogenic agents in it. All of these compounds are effective in reducing lipid deposition in differentiated 3T3-L1 preadipocytes. Moreover, oxypeucedanin, a potent anti-adipogenic coumarin, was found to regulate major players of adipogenesis, namely, PPAR-γ, SREBP-1, C/EBP-α, FABP-4, aP2, LPL and leptin expression in cells [66]. Water extract also showed similar inhibition of adipogenesis by inhibition of LPL secretion and induction of sortilin-related receptors mediated by C/EBPβ [76].

### 5.2. Activity in Prostatic Hyperplasia

Hydroalcoholic (70% Ethanol) extract of Ponciri Fructus significantly inhibited testosterone propionate induced prostatic hyperplasia in male Sprague Dawley rats. The extract reduced the weight of the prostate, the level of testosterone and dihydrotestosterone in the prostate tissue and serum of rats. This finding was attributed to diminished expression of proliferating cell nuclear antigen and increased endogenous antioxidant enzyme levels on subcutaneous administration of extract [32].

### 5.3. Anti-Inflammatory Activity/Anti-Allergic Effect/Immunoprotective Effects

The widespread exploitation of Ponciri fruits in traditional medicine as an anti-allergic agent in China, Korea, and Japan has also been supported by scientific studies. Lee et al., 2018, identified the anti-inflammatory activity of Ponciri Fructus on an in vitro model of Raw 264.7 cells [77]. The fruit extract reduced nitrous oxide production, matrix metallopeptidase, and proinflammatory cytokines (tumor necrosis factor alpha and interleukin-6) significantly in studied cells. The extract was also found to inhibit phosphorylation of ERK1/2, JNK, P38, MAPK, and nuclear translocation of P65 and NF-κB in LPS-stimulated cells [77]. The anti-allergic potential of this fruit was also demonstrated on an in vivo mouse model [78]. It was found that the administration of ponciri fruits together with Jiyutang decreased reactive oxygen intermediates (O_2_^−^ and H_2_O_2_) in neutrophil and macrophase, and reduced contact hypersensitivity without affecting spleen capacity to form rosette [78]. The dermal anti-allergic effect of fruit extract was also demonstrated in mice models showing decreased dermal thickness, epidermal skin, mast cell count, and IgE in serum. Furthermore, scratching frequency, nerve growth factor, and cytokine (IL-4, IL-6, IFN-γ and TNF-α) levels were also reduced significantly [79]. A recent study by Hwang et al. have shown that ethanol extract effectively inhibited septic shock and increased survival in LPS- and CLP-induced mice by inhibiting STAT1 signaling, supporting the anti-inflammatory activity of fruits [35]. Fruit extract consumption also controlled type-1 hypersensitivity reaction induced by anti-dinitrophenyl (DNP)-IgE and dinitrophenyl-human serum albumin in rats by inhibition of histamine secretion [80,81].

Isolation of active pharmacological constituents yielded a triterpenoid, hispidol A 25-methyl ether, with strong anti-inflammatory effects [82]. This compound inhibited bacterial infection-induced neuroinflammation in mice by inhibiting expression of proinflammatory cytokines [82]. These isolated compounds also showed significant anti-inflammatory activities in a carrageenan-induced paw edema model of mice and in vitro RAW 264.7 cells [83]. Rho et al. also isolated coumarin derivatives, imperatorin and phellopterin with strong anti-inflammatory effects [65].

### 5.4. Inhibition of Melanogenesis

Ethanol extract from immature fruits was found to significantly inhibit melanogenesis in B16F10 cells revealing its potential application as a cosmeceutical agent [84]. Extract hindered melanin biosynthesis by significantly reducing tyrosinase enzymatic activity and TRP-1 protein [84]. The potential was further extended to the anti-wrinkle activity of fruits in human dermal fibroblasts exposed to UVA radiation. They found that fruit extract fermented with *Ganoderma lucidum* inhibited matrix metalloproteinase 1 and also increased collagen biosynthesis in a dose dependent manner [85]. Interestingly, Son et al. isolated bis(2-methylheptyl) phthalate, a potent melanogenesis inhibitor from fruits. The isolated compound was highly effective with an IC_50_ value of 36.8 µM compared to standard kojic acid (IC_50_; 150 µM) in the B-16 melanoma cell line of mice [71].

### 5.5. Hypolipidemic Acactivity

Administration of Ponciri Fructus extract reduced total cholesterol, LDL cholesterol and triglyceride in the plasma and liver of hyperlipidemic rats while increasing indigenous antioxidant enzymes (catalase and glutathione peroxidase) activity [86]. A similar hypolipidemic activity was replicated by Ham et al. on triton WR-1339 induced hyperlipidemic rats showing with significant reduction of triglyceride, total cholesterol and LDL cholesterol on treatment with extract [87].

### 5.6. Anti-Cancer Activity

Methanolic extract of Ponciri Fructus was found to selectively induce apoptosis in glucose-deprived pancreatic cancer (PANC-1) cells [88]. The extract inhibited the expression of glucose-regulated protein 78 (GRP78; a marker that increases cell survival and decreases apoptotic potential during stress response) dose-dependently in pancreatic cells under hypoglycemic conditions [88]. Interestingly, triterpenoids pancastatin A and B were isolated from this fruit which showed similar selective cytotoxicity and reduction of GRP78 protein in PANC-1 cells [62]. The result was further supported by Kang et al. finding that aqueous decoction of Ponciri Fructus significantly increased natural killer cell activity responsible for antitumor immunity in vitro [89] and in vivo [90]. Sim et al. found that the extract treatment increased the survival of mice inoculated with sarcoma-180 cells by increasing natural killer cell activity [90]. Another imperative study on colorectal cancer cell line CT-26 showed that 70% methanolic extract from fruit significantly increased apoptosis by increasing mitochondrial autophagy [91]. The antiproliferative activity of fruit extract was also extended to the pituitary tumor cell line (GH3) [92]. The extract of fruit was also found to alleviate triple-negative breast cancer by increasing apoptosis through c-Jun(2)-terminal kinase and extracellular regulated kinase [93]. Furthermore, a triterpenoid, 25-Methoxyhispidol A, was isolated from this fruit showing anticancer activity in human hepatocarcinoma cells (SK-HEP-1) by cell cycle arrest in the G1 phase and by induction of apoptosis [51]. The extract also demonstrated dose-dependent cytotoxicity in HL-60 leukemia cells mainly increasing apoptosis by activation of caspase-3 and DNA fragmentation [50]. Interestingly, imperatorin and limonin isolated from seeds of Ponciri Fructus showed growth inhibition in liver cancer (SNU 449), and colon cancer (HCT-15) cells line. They caused cell cycle arrest and promoted apoptosis by regulating proapoptotic Bax and Bcl-2 expression dose dependently [53].

### 5.7. Cosmeceutical Uses

Ethyl acetate fraction of Ponciri Fructus inhibited the growth of nine different strains of methicillin resistant *Staphylococcus aureus*, a pathogen known to cause serious skin infections, with the minimum inhibitory concentration of 256 to 1024 µg/mL indicating its potential use as a cosmeceutical [94].

A case study on an 85 year old liver cirrhosis patient with an itching sensation on whole body showed topical administration of decoction obtained from Ponciri Fructus and Radix Lithospermi for 37 days improved erythema and pruritis [95].

### 5.8. Anti-Helicobactor pylori Effect (Gastroprotective)

Gastroesophageal reflux disease is one of the major health complications for many people around the world. Extract of Ponciri Fructus hastens gastric emptying and improves mucus secretion, ameliorating the effects. The activity of the fruit is due to the presence of bioactive compounds such as poncirin (by reducing gastric lesion induced by HCl), naringin (by preventing gastric ulcers), hesperidin (by improving delayed gastric emptying), neohesperidin (by stimulating mucus secretion) [48]. Administration of poncirin, a major flavonoid of Ponciri Fructus, after metabolism gets converted into ponciretin and inhibits the growth of *Helicobactor pylori* in the intestine with a MIC of 10–20 μg/mL [33].

### 5.9. Prokinetic Effect

The serotonin receptor (5-HT receptor) of the gastrointestinal tract is known to play a key role in intestinal motility. Intestinal cells of Cajal in the murine model have three of those serotonin receptor subtypes making it a key target for studying the influence on gastric movements [54]. The methanolic extract of Ponciri Fructus modulated pacemaker potential interstitial Cajal cells of the small intestine by 5-HT3, 5-HT4 receptor mediated channels. The extract increases the influx of Na^+^ and Ca^2+^ externally, and Ca^2+^ from the internal store proportional to mitogen-activated protein kinase levels increasing gastric peristalsis [54]. The hexane extract of Ponciri Fructus was found to stimulate longitudinal muscles of the distal colon in rats mediated by acetylcholinergic M2 and M3 receptors, supporting its prokinetic activity [96]. In vivo and in vitro study by Choi et al. showed that hexane extract from the fruit significantly and dose-dependently increased longitudinal muscle contraction in the distal part of colon [96]. The prokinetic activity of the aqueous extract was also identified by Lee et al. in mice showing an acceleration of gastric contents throughout the gastrointestinal tract [56].

A clinical trial on 25 patients with neurogenic bowel due to spinal cord injury, fed with 800 mg of water decoction of Ponciri Fructus twice a day for 2-week periods, showing improved constipation resulting from short colon transit and improved stool morphology [97]. None of the subjects showed serious side effects except soft stool (2 people) and diarrhea (5 people) [97].

### 5.10. Hepatoprotective Activity

Ponciri Fructus contains hepatoprotective moieties isoimperatorin, which significantly inhibited aflatoxin B1-induced cytotoxicity in H4IIE cells by increasing endogenous glutathione and cytochrome p450 enzymes [36].

### 5.11. Tick Repellent Effect

Decoction of Ponciri Fructus showed tick-repellent properties towards *Boophilus microplus* and *Haemaphysalis bispinosum* species [98].

### 5.12. Effect on Acute Pancreatis

Pretreatment with Ponciri Fructus at the doses of 200 and 400 mg/kg ameliorated cerulein-induced acute pancreatitis in mice. The extract reduced pancreatic damage, myloperoxide level, and serum amylase in studied mice [99].

### 5.13. Neuroprotective Effects

25-Methoxy hispidol A isolated from fruits showed a significant neuroprotective effect in the anxiety and depression model of mice induced by bacterial infection. The compound demonstrated significant improvement in different behavioral tests, endogenous antioxidant enzyme levels, neurodegeneration of the hippocampus, and degree of inflammation [82].

### 5.14. Antiviral Activity

Ethanol extract of Ponciri Fructus inhibited the oseltamivir-resistant strain of influenza virus grown in Madin-Darby canine kidney cells. The extract was reported to work by altering the cellular penetration pathway of the virus into the cells [100].

Table 3 below shows list of bioactive compounds and their bioactivities observed in literature.

## 6. Polyherbal Formulations Containing Ponciri Fructus

### 6.1. Sam-Chul-Kun-Bi-Tang

Sam-chul-kun-bi-tang constitutes fourteen different herbal agents among which Ponciri Fructus covers 1/15th part by weight. This is widely used in Korean traditional medicine for the treatment of gastroptosis, chronic gastritis, and gastric ulcer. Several pharmacological activities have been identified in this formula including immunoregulatory activities, anti-inflammatory activity, and gastroprotective activity [171]. The formulation was found to modulate the pacemaker activity of interstitial cells of Cajal to control intestinal motility [172]. Yoo et al. also identified in vitro anti-adipogenic activity in 3T3-L1 preadipocytes and antioxidant activity in DPPH and ABTS free radical scavenging assays [37].

### 6.2. Jeechool-Whan

Jeechool-Whan is a traditional Korean medicine for the treatment of allergic diseases by strengthening spleen and stomach functions. This is due to the belief in Eastern Medical Theory that all diseases are the result of altered spleen and stomach functions. This formulation is made by mixing Ponciri Fructus and Atractylodis Rhizoma in a ratio of 1:2. A study on female BALB/c mice model of allergic rhinitis induced by ovalbumin showed that this formulation inhibited itching, levels of IgE, histamine, and inflammatory cytokines inhibiting the allergic response [42].

### 6.3. Mahwangyounpae-Tang

This herbal formula contains 22 different herbal extracts and has been used for ages to treat asthma and other respiratory diseases. Ponciri Fructus constitutes 1/25th part by weight in this formula. Park et al. found that an aqueous extract of this polyherbal formula is safe up to a 400 mg/kg dose in mice [38]. However, the extract was found to be effective at the low dose of 30 mg/kg in ovalbumin-induced asthmatic mice. The extract preserved trachea architecture by reducing inflammation [173]. The anti-asthmatic activity of this formulation was also supported by antibacterial effects and synergism with standard antibiotics [174].

### 6.4. Cheonggan (CGX)

Traditional Korean herbal medicine, CGX, is made by mixing 13 different herbs, in which Ponciri Fructus constitutes 1/19th part by weight. It is a famous formula for the treatment of patients with chronic hepatic diseases including hepatitis-B, fatty liver and alcoholic hepatitis [39]. Traditional use of this formula was also demonstrated in different clinical and experimental models of liver toxicity. This formula was also found to work by its antioxidant and immune regulatory potential [39].

### 6.5. Ojeok-San

This is one of the long used traditional Korean medicines prepared by mixing 17 different herbal agents including Ponciri Fructus (almost 1/18th part by weight). This formulation is prescribed for fever, rheumatism, inflammation, and hyperlipidemia [42].

## 7. Safety Issues

This fruit has been used for ages in traditional medicine safely; however, recent scientific studies also support those findings. A clinical trial on 25 patients with neurogenic bowel dysfunction were fed with 800 mg of water decoction obtained from Ponciri Fructus twice a day for 2-week periods. Which was found to be safe with minor side effects in a very low population (7 Patients: soft stool (2 people) and diarrhea (5 people)) [97]. Ojeok-San was also found safe in ICR mice up to a dose of 5000 mg/kg without noticeable toxicity [175]. Research showed no toxicity associated with Samchulgeonbi-tang consumption at the dose of 2000 mg/kg in ICR mice [176].

**Figure 3:**
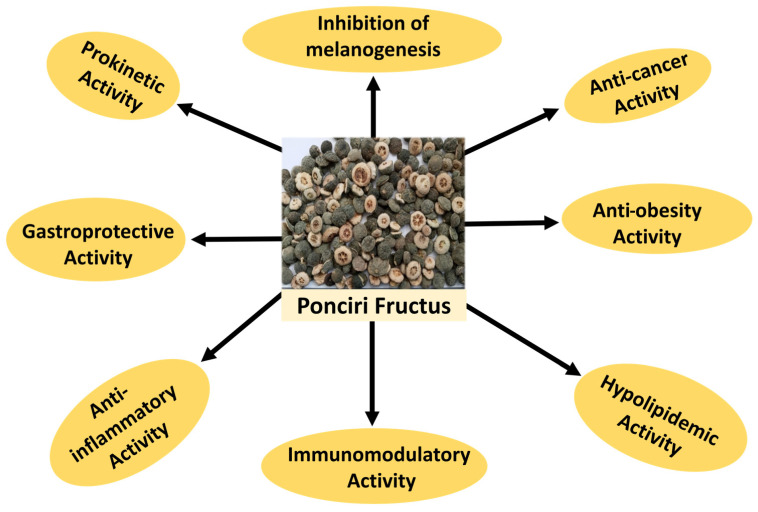
Summary of pharmacological activities of Ponciri Fructus.

Figure 3 below shows summery of biological activities shown by Ponciri Fructus extract on different studies model.

## 8. Conclusion and Future Recommendation

In summary, this review article abridged the ethnomedicinal uses, bioactive constituents, and biological activities of Ponciri Fructus, a popular nutritional and medicinal plant used extensively in East Asian countries. Different books, traditional monographs, and scientific articles have mentioned Ponciri Fructus as a valuable nutritional component and effective folk remedy. Despite this, comprehensive information about collection time and techniques, processing methods, formulation technique, adequate dose, duration of medication, and possible toxicity have not been explained in detail yet. Therefore, there should be future clinical practice and investigation of Ponciri Fructus. Meticulous investigation of bioassay-guided isolation is mandatory because it is a very difficult task to optimize the biological activity of a multi-component complex herbal mixture to a targeted single bioactive molecule, because it possesses several classes of bioactive constituents. Moreover, the quantitative determination of major bioactive compounds present in Ponciri Fructus will be a key step to standardize the pharmacopeial standards for the crude drug, its extract, and possible formulations. In addition, great emphasis on the examination of possible toxicity in the human model and the study of pharmacokinetic parameters is recommended strongly. Moreover, sophisticated scientific investigations involving animal models and the discovery of the mechanism of action of bioactive molecules at the molecular level are very much crucial in future works.

## Figures and Tables

**Table 1:** Chemical composition of essential oils isolated from the fresh peel of *P. trifoliata* fruits [69].

Compounds	Relative Amount (%)
Limonene	41.73
Myrcene	15.68
(E)-β-Ocimene	5.05
α-Phellandrene	4.11
β-Pinene	3.95
*trans*-Caryophyllene	3.59
Ethyl hexanoate	2.22
Ethyl octanoate	2.12
*p*-Cimene	1.5
*α*-Pinene	1.19
Sabinene	1.17
*β*-Farnesene	1.16
(*E*,*Z*)-Farnesol	1.02
Hexyl butanoate	0.98
*δ*-Cadinene	0.73
α-Humulene	0.72
Ethyl decanoate	0.72
*γ*-Terpinene	0.70
Linalool	0.69
Nerol	0.65
*β*-Elemene	0.59
Geranyl acetate	0.56
Terpinolene	0.54
Geraniol	0.52
Germacrene B	0.51
*p*-Mentha-1,3,8-triene	0.46
Ethyl laurate	0.46
Spathulenol	0.44
(*E*)-Nerolidol	0.42
Decanal	0.42
Pentacosane	0.42
Ethyl stearate	0.35
Eicosane	0.35
Neryl acetate	0.34
Heptacosane	0.32
Ethyl myristate	0.31
*α*-Terpineol	0.30
*α*-Cubebene	0.28
Myristic acid	0.26
Ethyl linoleate	0.23
Dodecanal	0.22
*γ*-Cadinene	0.20
Octadecane	0.14
Nonadecane	0.12
Nonacosane	<0.1
Nonanal	<0.1
Terpinen-4-ol	<0.1
α-Terpinene	<0.1

**Table 2:** Quantitative analysis of the chemical constituents from the fresh fruit juice and seed extract of *P. trifoliata* by using HPLC [69].

Identified Compounds	Fresh Fruit Juice (µg/mL)	Seed Extract (µg/g of Dry Extract)
**Flavanone aglycones**		
Hesperetin	55.13	Not detected
Naringenin	28.65	Not detected
**Flavanone-*O*-glycosides**		
Didymin	78.83	156.42
Hesperidin	129.33	Not detected
Naringin	115.79	Not detected
Narirutin	75.73	37.62
Neohesperedin	32.75	80.12
Poncirin	49.37	Not detected
**Flavone aglycones**		
Rhamnetin	0.37	Not detected
Quercetin	0.76	Not detected
**Flavone-*O*-glycoside**		
Rutin	1.85	Not detected
**Phenolic acids**		
Caffeic acid	18.46	32.85
Chlorogenic acid	112.54	Not Detected

**Table 3:** Bioactive chemical constituents isolated from Ponciri Fructus and their pharmacological activities.

Bioactive Compound	Pharmacological Activities
Poncirin	Anticancer [101,102], antidiabetic [103], hepatoprotective [104,105], analgesics [106], anti-inflammatory [106,107], attenuation of colitis [108], anti-bacterial, anti-adipogenic [109], neuroprotective [110], suppression of osteoclast differentiation [111]
Neoponcirin (didymin)	Anticancer, neuroprotective, anxiolytic, antinociceptive hepatoprotective, and cardioprotective, anti-inflammatory activities [112], antidiabetic [113]
Naringin	Anticancer, antioxidant, anti-inflammatory, neuroprotective, increased bone regeneration, genoprotective, amelioration of metabolic syndrome [114], hepatoprotective, cardioprotective, gastroprotective, immuno-promotive [115], hypocholesterolemic [116], renoprotective [117], antibacterial [118]
Hespiridin	Antioxident, anti-inflammatory, anticancer, antiviral, cardioprotective, neuroprotective, antibacterial, radioprotective, wound healing [119], antidiabetic [120]
Neohesperidin	Neuroprotective, anti-inflammatory, antidiabetic, antimicrobial, anticancer, gastroprotective, cardioprotective, hepatoprotective, anti-obesity [121], antioxidant [122], increased glucose uptake [123]
Sinensetin	Antiangiogenetic, anticancer, anti-dementia, anti-inflammatory, antimicrobial, anti-obesity, antidiabetic, antioxidant, anti-trypanosomal, diuretic, hypolipidemic, vasorelaxant [124], neuroprotective [125], alleviation of age-related muscle loss [126], gastroprotective [127]
Nobiletin	Anticancer, anti-obesity, antioxidant, memory enhancing, chondroprotective, anti-tuberculosis, hepatoprotective, cardioprotective, anti-hypertensive, immunoenhancing, gastroprotective, antiviral [128], amelioration of metabolic disorders [129], anti-aging [130]
Hesperetin	Anticancer, cardioprotective, regulation of carbohydrate and lipids metabolism, osteoprotective, anti-asthmatic, radioprotective, regulation of melanogenesis, anti-inflammatory [131], antidiabetic [132], mitigation of heavy metals toxicity [133], neuroprotective [134]
Narirutin	Anticancer, neuroprotective, anti-inflammatory, antidepressant, hepatoprotective, antioxidant, anti-adipogenic, immunomodulatory, antiallergic [135], analgesics [136], antidiabetic [137]
21β-Methylmelianodiol	Anti-inflammatory [138]
Hispidol β 25-methyl ether	Anti-inflammatory [83], neuroprotective [82]
Hispidol A 25-methyl ether	Neuroprotective [139]
21α-Methylmelianodiol	Anti-inflammatory [138], anticancer [140]
Pancastatin A and B	Anticancer [62]
25-Methoxyhispidol A and B	Neuroprotective [139], anticancer [51], anti-inflammatory [83]
Methoxsalen	Vitiligo management [141]
Imperatorin	Neuroprotective, anticancer, anti-inflammatory, antihypertensive, antibacterial, antiviral, anticoagulant [142], antioxidant [143], antiallergic [144], anti-psoriatic [145], regulation of melanogenesis [146], antidepressant [147]
Isoimperatorin	Anticancer [148,149], antiviral [150], anti-asthmatic [151], antibacterial [152], anti-adipogenic [153], anti-inflammatory [154], alleviation of osteoarthritis [155]
Phellopterin	Wound healing, anti-inflammatory [156], anticancer [157], anti-adipogenic [66]
Auraptene	Neuroprotective, antioxidant, anticancer, inhibition of platelet aggregation, hepatoprotective, antibacterial [158], cardioprotection [159], anti-inflammatory [160], melanogenesis inhibition [161]
Umbelliferone	Antibacterial, antifungal, antioxidant, antidiabetic, anticancer, molluscicidal [162], antiviral [163], antidiarrheal [164], antiallergic [165], anti- Alzheimer [166]
Oxypeucedanin	Antiallergic, antiarrhythmic, anticonvulsant, antifeedant, antigenotoxic, ant inflammatory, antimalarial, antimicrobial, antioxidant, antiproliferative, antiviral, calcium antagonistic, cytotoxic, insecticidal, phytotoxic [167], anti-adipogenic [66]
Oxypeucedanin methanolate	Antioxidant, antibacterial [168], antimalarial [169], anti-inflammatory [170]

## Data Availability

Not applicable.

## Sample Availability

Samples of the compounds are not available from the authors.
